# Multilayer Fused Correntropy Reprsenstation for Fault Diagnosis of Mechanical Equipment

**DOI:** 10.3390/s24186142

**Published:** 2024-09-23

**Authors:** Qi Deng, Guanhui Zhao, Weixiong Jiang, Jun Wu, Tianjiao Dai

**Affiliations:** 1 School of Naval Architecture and Ocean Engineering, Huazhong University of Science and Technology, Wuhan 430074, China; dengqi@hust.edu.cn (Q.D.); jiangweixiong@hust.edu.cn (W.J.); tianjiaodai@hust.edu.cn (T.D.); 2 College of Computer Science and Technology, Zhejiang University, Hangzhou 310027, China; 12321305@zju.edu.cn; 3 China Ship Development and Design Center, Wuhan 430064, China

**Keywords:** mechanical equipment, fault diagnosis, multilayer fused correntropy representation

## Abstract

Fault diagnosis is vital for improving the reliability and safety of mechanical equipment. Existing fault diagnosis methods require a large number of samples for model training. However, in real-world environments, mechanical equipment usually operates under healthy conditions during most of its service life, resulting in a scarcity of fault samples. To solve this problem, a novel multilayer fusion correntropy representation method combined with a support vector machine is proposed for the fault diagnosis of mechanical equipment. First, the monitoring signal is expanded into multilayer signal components using wavelet packet decomposition. Then, the correlation between the signal components of each layer is expressed by correntropy, and the corresponding correntropy matrix is constructed. After performing the matrix logarithm operator, all correntropy matrices composed of correntropy values are fused into a vector, which is viewed as a feature of the signal. Finally, a support vector machine is established using small samples to realize fault classification. The effectiveness of the proposed method is validated on four public datasets. The results indicate that compared with other methods, the proposed method has advantages in terms of diagnosis accuracy and noise immunity ability.

## 1. Introduction

Fault diagnosis of mechanical equipment, such as gears and bearings, can reduce maintenance costs and ensure operation security. The development of fault diagnosis technology has become a key direction in industrial applications [1,2,3,4].

A large number of machine learning methods have been widely used for mechanical fault diagnosis. These methods usually include end-to-end deep learning (DL)-based methods and traditional machine learning (ML)-based methods. End-to-end DL-based methods have garnered considerable attention in the academic field because they have the ability to extract representative information from input samples automatically. Widely used DL-based methods include convolutional neural networks (CNN) [5,6,7], recurrent neural networks (RNN) [8], and transformers [9]. For example, Huang et al. [10] proposed a multiscale CNN based on wavelet packet decomposition (WPD), which effectively diagnosed multiple faults in a wind turbine gearbox. Qin et al. [11] proposed a large model based on a dense connection network with depthwise separable convolution. Numerous datasets from various rotating machinery were used for model training; thus, the model has strong feature extraction and generalization capabilities. Zhang et al. proposed a dual path convolutional with attention mechanism (DCA) and bi-directional gated recurrent unit (DCA-BiGRU) network, which was validated on bearing and gear datasets. Yan et al. [12] proposed a lightweight fault diagnosis framework based on a separable multiscale convolution and broadcast self-attention mechanism, which diagnosed different faults in the planetary gearbox, spur gear, and bearing.

Although these DL-based fault diagnosis methods have yielded promising results, they have limited applications in the engineering field. They have deep hierarchical networks with a black-box problem, which always puzzles maintenance engineers and technicians. In other words, it is difficult for us to understand the decision-making process of those methods. However, traditional ML-based methods rarely suffer from this problem.

Traditional ML-based methods consist of two essential parts: a feature extraction method and a classifier. Feature extraction methods include statistical features [13], slow feature analysis [14], and wavelet packet decomposition [15]. Typical classifiers include ridge regression, support vector machine (SVM), and random forest. Feature extraction methods transform high-dimensional signals into low-dimensional feature vectors. Then, the feature vector is inputted into the classifier to diagnose different fault types. As an example, Li et al. [16] constructed a feature-fusion covariance matrix (FFCM) with time and frequency domain statistical features from multi-sensor signals for feature extraction, and then built multi-Riemannian kernel ridge regression (MRKRR) for fault classification. Su et al. [17] fused singular value manifold features from multisource sensors for feature extraction and developed optimized SVMs for fault classification. Chai et al. [18] utilized slow feature analysis to establish static and dynamic analysis nodes for feature extraction and used improved random forest for fault classification.

Most of the methods above usually adopt human priori knowledge or signal processing methods for feature extraction and use classifiers with solid mathematical theories. Thus, traditional ML-based fault diagnosis methods are more conducive to engineering applications than DL-based methods. However, traditional ML-based methods are severely limited in building diagnostic models due to the lack of available samples. In many cases, mechanical equipment usually operates in a healthy condition during its service life. In addition, regular maintenance is generally performed to avoid unexpected faults and ensure reliable and safe operation. Therefore, fault samples are scarce in real-world environments [19,20]. To address small sample limitations, Wang et al. [21] proposed a refined composite multiscale phase entropy (RCMPhE) to extract entropy features and introduced a bonobo optimization SVM for fault classification. Yang et al. [22] developed a hierarchical symbol transition entropy (HSTE) and a 2-D-extreme learning machine for fault classification. Feng et al. [23] proposed a temporal local correntropy representation (TLCE) method for feature extraction and adopted an SVM to achieve accurate fault classification.

Although these methods can overcome the sample scarcity issue, they may still have room for improvement in terms of diagnostic performance and data requirements. For example, TLCE first divides the monitoring signal into several segments and then uses the sliding window to generate samples in the direction of the time sequence. Finally, the correntropy between the segments in a sample is computed and combined into a correntropy matrix, which is viewed as the extracted feature of the sample. The main drawback of TLCE is that the excellent quality of the extracted features depends on a sufficient number of segments in the sample. However, the greater the number of segments, the greater the sample length. This means that more training data are still necessary. To overcome the above drawback, a new multilayer fusion correntropy representation (MFCE) is proposed for the fault diagnosis of mechanical equipment with small sample sizes. Similar to TLCE, the core purpose of MFCE is to extract highly discriminative features to achieve accurate fault diagnosis. Unlike TLCE, MFCE uses only a segment as a sample and decomposes it into multilayer signal components based on WPD. Then, the correntropy values among the signal components of each layer are calculated and fused into the feature of the sample. Thus, MFCE requires less training data than TLCE. The main contributions of this paper are as follows:

1. A new fault diagnosis method is proposed using MFCE combined with an SVM for mechanical equipment under noise interference scenarios.
2. An MFCE is designed to extract representative features from signals when only a small number of samples are available.

The rest of the paper is organized as follows. Section 2 presents the methodology. Section 3 presents the experimental results and analysis. Section 4 concludes this paper.

## 2. Methodology

**Figure 1:**
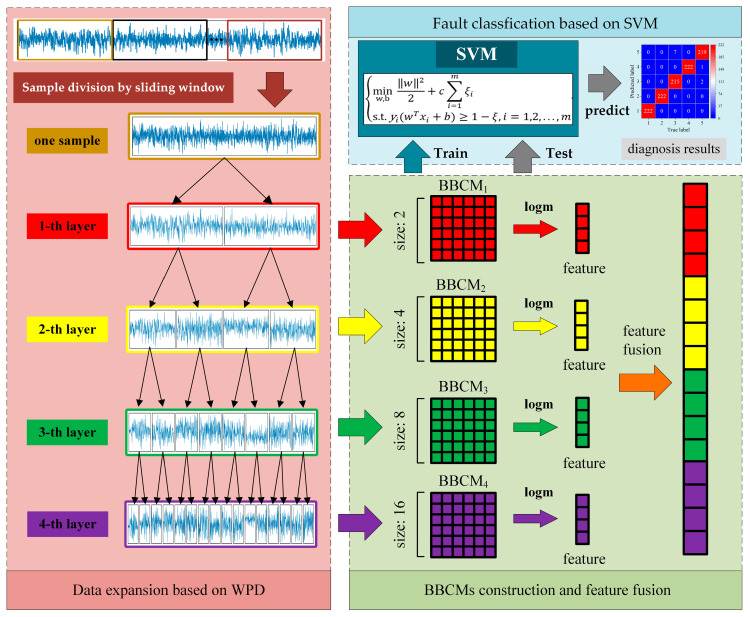
Flowchart of fault diagnosis based on the proposed MFCE combined with SVM.

The proposed MFCE consists of two procedures: data expansion based on WPD and between-components correntropy matrices (BBCMs) construction and feature fusion, which are shown in Figure 1. In detail, in the first procedure, the monitoring signal collected from mechanical equipment is divided into a series of samples by a sliding window. Then, each sample is expanded into multilayer signal components by WPD. In the second procedure, BBCMs from all layers are calculated, and the matrix logarithm (logm) operator is applied to all BBCMs. Finally, the correntropy values from the BCCMs are fused into a vector, which serves as the feature of the sample.

After feature extraction, all samples are randomly selected to form a training set and a testing set. During the training stage, the SVM is modeled with the training set. During the test stage, the testing set is inputted into the well-trained SVM to obtain the diagnosis results.

### 2.1. Data Expansion Based on WPD

As a signal analysis method, WPD is introduced to decompose the monitoring signal into multiple signal components with the same bandwidth but different center frequencies. Compared with wavelet decomposition, its advantage lies in its capability to decompose high-frequency portions of the signal with no redundant or missing information. For complex industrial environments, it is well suited for processing unsteady mechanical vibration signals with high-frequency characteristics and intense background noise [24].

In WPD, each node (*k*, *j*) on the wavelet tree represents a vector space consisting of a series of standard orthogonal base $W_{k}^{j}$. The standard orthogonal bases are denoted as ${\{\psi }_{k}^{j}(q-2t)\},q\in Z$, *Z* is the integer set. Based on the recursive relationship, two wavelet packet orthogonal bases of a child node are defined as:

(1) $$\begin{array}{l} \psi_{k}^{2j-1}(t)=\sum_{q}h(q)\psi_{k}^{j}(q-2t)\\ \psi_{k}^{2j}(t)=\sum_{q}g(q)\psi_{k}^{j}(q-2t) \end{array}$$

where h(⋅) and g(⋅) are the high-pass and low-pass filters in multiresolution analysis, respectively.

The orthogonal spaces $W_{k}^{2j}$ and $W_{k}^{2j-1}$ are defined as the components $S_{k}^{2j}$ and $S_{k}^{2j-1}$. The recursive division defines a wavelet packet space tree where each parent node is divided into orthogonal subspaces:

(2) $$W_{k}^{j}=W_{k}^{2j}{⨁\;W}_{k}^{2j-1}$$

Then, all components can be defined by the following recursive relationship:

(3) $$\left\{\begin{array}{l} S_{0}^{0}=S\left(q\right)\\ S_{k}^{j}(q)=S_{k}^{2j-1}(q)+S_{k}^{2j}(q)\\ S_{k}^{2j-1}(q)=\sum_{q}S_{k-1}^{j}(q)h(q-2t)\\ S_{k}^{2j}(q)=\sum_{q}S_{k-1}^{j}(q)g(q-2t) \end{array}\right.$$

where the $S_{0}^{0}$ represents the signal corresponding to node (0, 0) on the wavelet tree, i.e., the original signal.

Finally, the components of the *k*th layer can be represented by the following matrix:

(4) $$S_{k}\left(q\right)=[S_{k}^{0}\left(q\right),S_{k}^{1}\left(q\right),\ldots ,S_{k}^{2^{k}-1}\left(q\right)]$$

When signals are decomposed by WPD, the choice of basis functions and the number of decomposition layers are critical for subsequent correntropy calculations.

### 2.2. Between-Components Correntropy Matrices Construction and Feature Fusion

Correntropy is a correlation measurement method that combines information-theoretic learning and kernel functions [25]. It is mainly used to measure non-Gaussian and nonlinear dependencies in the data’s statistical properties. Here, correntropy is used to measure the correlation between the signal components from different layers. If there are two independent variables x and y, then the correntropy between them can be expressed as:

(5) $$C_{\sigma }\left(x,y\right)=E\left[k_{\sigma }\left(x,y\right)\right]=\int k_{\sigma }\left(x,y\right)dF_{x,y}\left(x,y\right)$$

where $dF_{x,y}(x,y)$ and E[⋅] denote the joint probability density function of (x,y) and the expectation function. $k_{\sigma }(\cdot )$ denotes the kernel function.

The default $k_{\sigma }(x,y)$ is the Gaussian kernel, denoted as:

(6) $$k_{\sigma }(x,y)=\frac{1}{\sqrt{2\pi }\sigma }\exp\left(-\frac{{\left(x-y\right)}^{2}}{\sqrt{2\sigma^{2}}}\right)$$

where σ is the kernel length.

For engineering applications, signal x and y of length N are limited, so the correntropy can be expressed as

(7) $$C_{\sigma }(x,y)=E\left[k_{\sigma }(x,y)\right]\approx \frac{1}{N}\sum_{i=1}^{N}k_{\sigma }(x,y)$$

The correntropy with a Gaussian kernel is symmetric, and its Taylor expansion is

(8) $$C_{\sigma }(x,y)=\frac{1}{\sqrt{2\pi }\sigma }\sum_{n=0}^{\infty }\frac{(-1{)}^{n}}{{\left(2\sigma \right)}^{n}n!}E\left[{\left(x-y\right)}^{2n}\right]$$

Compared with lower-order statistics (e.g., mean square error), the correntropy with a Gaussian kernel is the sum of the even-order moments of the difference between signal x and y. Thus, it can provide information on higher-order moments. Moreover, it provides a more generalized similarity measure than the correlation function; therefore, it is considered a generalized correlation function.

Based on the signal components and Equation (7), the between-components correntropy matrix of the *k*th layer can be constructed:

(9) $$BCCM_{k}=\left\{C_{\sigma }(S_{k}^{i},S_{k}^{j})\right\},i,j=1,2,...,2^{k}-1$$

Since the main diagonal of the BCCM represents the correntropy of the component and itself, its values are all 1. The non-main diagonal elements are the correntropies between different components; therefore, their values are between 0 and 1. The values with the main diagonal as the symmetry axis have the same magnitude. The BCCM is characterized by the fact that it contains correlation information between multiple components with different center frequencies, which can effectively express the correlation between the internal scales of the monitoring signals.

BCCMs are symmetric positive definite matrices that lie in the Riemannian manifold space. Before feature fusion, a matrix logarithmic operation is used to transform it from the Riemannian manifold space to the Euclidean metric space. The matrix logarithm operator is denoted as

(10) $$\mathrm{logm}\left(A\right)=Udiag\{\log\{diag\Lambda \}\}U^{T}$$

where A is represented by the eigenvalue decomposition $A=U\Lambda U^{T}$. U is the characteristic matrix, Λ is the diagonal matrix containing the eigenvalues, and T is the matrix transpose operator.

After that, the obtained 2-dimensional BCCMs are reshaped into 1-dimensional vectors. Finally, the vectors from all layers are concatenated into a feature vector, denoted as

(11) $$F=[V_{1}⊞V_{2}⊞\cdots ⊞V_{K}]$$

where $V_{k}=logm\left({BCCM}_{k}\right),k=1,2,\ldots ,K$. K is the number of decomposition layers. ⊞ is the vector concatenation operator.

It is worth noting that the sizes of the constructed BCCM from different layers are different. The size of ${BCCM}_{k}$ is $2^{k}\times 2^{k}$, so the number of features extracted by MFCE is $4^{1}+4^{2}+\ldots +4^{K}=4(4^{K}-1)/3$.

### 2.3. Fault Classification Based on SVM

A support vector machine (SVM) has a significant advantage in solving small sample sizes and nonlinearity issues. Not only that, it can overcome dimensionality catastrophe, suppress model overfitting, and improve computational efficiency. For a training set with *m* samples ${\left\{\left(x_{i},y_{i}\right)\right\}}_{i=1}^{m}$, samples $x_{i}$ are labeled as one of two categories $y_{i}\in \{+1,-1\}$. The SVM represents the samples as points in space and finds the optimal hyperplane $w^{T}x+b=0$, (w and b are the weight vector and bias), such that there is an interval as wide as possible between samples of different categories. The broadest interval can be transformed into a minimum $w^{T}w$, and using the positivity and negativity of $y(w^{T}x+b)$ to determine the category of the sample:

(12) $$\left\{\begin{array}{l} \underset{w,b}{\min}\frac{{\left\|w\right\|}^{2}}{2}+c\sum_{i=1}^{m}\xi_{i}\\ s.t.\;y_{i}(w^{T}x_{i}+b)\geq 1-\xi ,\;i=1,2,...,m \end{array}\right.$$

where the penalty factor c is finite, which means that it allows some samples to dissatisfy the constraint, while c is infinite, which means that it requires all samples to satisfy the constraint. ξ is the slack variable in the soft interval concept.

Based on the Lagrangian function, a dual transformation of Equation (12) yields

(13) $$\left\{\begin{array}{l} \underset{\alpha }{\max}\sum_{i=1}^{m}\alpha_{i}-\frac{1}{2}\sum_{i,j=1}^{m}\alpha_{i}\alpha_{j}y_{i}y_{j}\phi (x_{i}{)}^{T}\phi (x_{j})\\ s.t.\;0\leq \alpha_{i}\leq G,\sum_{i=1}^{m}\alpha_{i}y_{i}=0,\;i=1,2,...,m \end{array}\right.$$

where $\alpha_{i}{,\alpha }_{j}$ are the Lagrange coefficients. ϕ(⋅) is the kernel function that maps samples from the original space into the high-dimensional kernel space. Here, the SVM with the precomputed mode is adopted for a fair comparison with the TLCE. In detail, the linear kernel matrix is computed before the samples are fed into the SVM.

During the test phase, the SVM can map a new sample into the kernel space and predict its category label based on which side of the hyperplane it falls on.

## 3. Case Studies

This section verifies the effectiveness of the proposed MFCE on four public datasets of mechanical equipment and analyzes its superiority by comparing it with other methods with small sample sizes. Subsequently, the noise robustness and critical parameters of the MFCE are analyzed. Ablation experiments are implemented to validate its rationality.

### 3.1. Dataset Introduction

**Figure 2:**
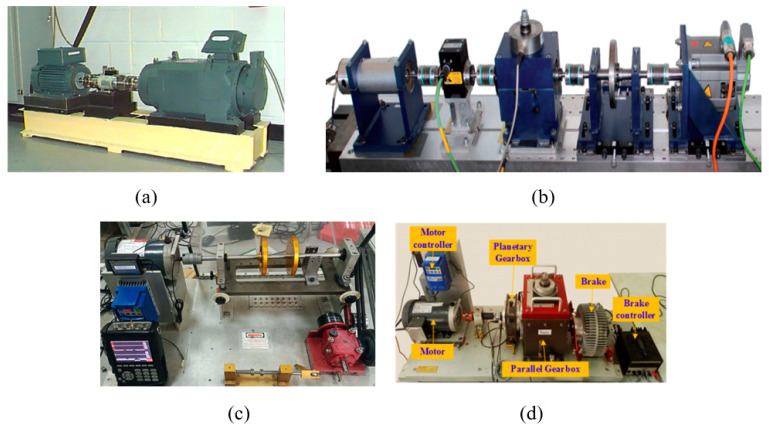
Fault simulation test bench: (**a**) CWRU dataset. (**b**) PU dataset. (**c**) SQ dataset. (**d**) Gearbox dataset.

The fault simulation test benches for the four datasets are shown in Figure 2. For simplicity, only some essential information is depicted, as follows:

#### 3.1.1. Case 1: CWRU Dataset

Bearing health states of the CWRU dataset [26] include normal, inner ring fault, rolling element fault, and outer ring fault. To simulate pitting faults of rolling bearings with various degrees, electrical discharge machining (EDM) was used to generate faults with diameters of 0.007 inches, 0.014 inches, and 0.021 inches, respectively. This dataset includes three bearing fault types, each of which consists of the three inches described above. Thus, there are ten types of class labels. The vibration signals with a sampling frequency of 12 kHz and a rotational speed of 1730 r/min were used in this paper.

#### 3.1.2. Case 2: PU Dataset

Bearing health states of the PU dataset [27] include normal, inner ring faults, and outer ring faults. Manual damage methods adopted for this dataset included EDM, drilling, and manual electronic engraving. The combination of different manual damage methods and different damage levels resulted in a sum of nine types of class labels. The sampling frequency used in this experiment was 64 kHz. The vibration signals with a rotational speed of 900 r/min were adopted.

#### 3.1.3. Case 3: SQ Dataset

A comprehensive fault simulation test bench from Spectrum Quest company was used to simulate motor bearing faults [28]. The sampling frequency was 25.6 kHz, and the duration of each acquisition was 15 s, including the complete acceleration and deceleration process from static gradually accelerating to 3000 r/min, then remaining stable, and finally gradually decelerating to zero. In this experiment, 15 s vibration signals under a steady speed were intercepted and used. The bearing health states include the normal, inner ring fault, and outer ring fault states. The three different damage levels are combined into seven types of fault labels.

#### 3.1.4. Case 4: Gearbox Dataset

The Gearbox dataset [29] is collected from the power system dynamics simulator by Southeast University. The sampling frequency of the vibration signals in this dataset is 5.12 kHz, and the rotational speed is 1200 r/min. The Gearbox dataset consists of five gear health states, i.e., the normal, broken tooth, wear, gear root fault, and gear surface fault.

Before the validations of the proposed MFCE are carried out, its parameters need to be determined. MFCE has four key parameters, which are the number of decomposition layers *K*, the wavelet basis function, sample length *L*, and the kernel length σ of correntropy. *K* and the wavelet basis function are temporarily set to 4 and Daubechies 8 (db8). For a better comparison, the kernel size of MFCE is consistent with that of TLCE, which is 0.15, 0.2, 0.6, and 0.005 for the CWRU, PU, SQ, and Gearbox datasets, respectively. To reduce the required training data, the sample length of MFCE is consistent with the segment length of TLCE, which is 1024, 6000, 3000, and 4600 for the CWRU, PU, SQ, and Gearbox datasets, respectively.

Samples were intercepted directly from the signals using a sliding window. The neighboring samples are without any overlap. The total sample size and other information about the four datasets are listed in Table 1. In the following experiments, the training samples were randomly selected from all samples, and the remaining samples were used for testing. To reduce the impact of randomness, the experiment was repeated 50 times, and the average accuracy was recorded.

### 3.2. Experimental Results and Discussion

To verify the effectiveness of the proposed MFCE under small sample sizes, comparison experiments with the TLCE were first implemented. Table 2 shows the accuracies and required data of MFCE and TLCE in the one-shot setting. One shot means that only one sample per category is involved in model training. The results show that the average accuracies of MFCE on the CWRU, PU, SQ, and Gearbox datasets are 98.60%, 94.98%, 96.32%, and 98.21%, respectively. Compared with TLCE, MFCE improves by 0.51%, 1.71%, 4.67%, and 0.73%, respectively. Importantly, MFCE requires only data points from one segment to achieve these accuracies. Thus, the data requirements are reduced by 92.31%, 95.65%, 93.75%, and 96.67% compared to TLCE, respectively.

It can be concluded that MFCE has a significant advantage in terms of diagnostic accuracy and data requirements. The reason for achieving good performance is that extracted features by MFCE are highly discriminative, and the SVM controls the degree of model fitting well through a penalty factor under small sample sizes. Meanwhile, MFCE mines the interior correlation (i.e., the correlation between the decomposed signal components) from the vibration signals, while TLCE mines the data correlation from the temporal dimension. Therefore, MFCE requires less training data than TLCE. Due to shorter testing samples, MFCE is more conducive than TLCE to refined fault diagnosis in real-world applications.

To further validate the superiority of the proposed MFCE, feature extraction methods (including wavelet packet energy (WPE), wavelet packet energy entropy (WPEE), and minimally random convolutional kernel transform (MiniRocket) [30]), and deep learning-based methods (ensembled transformer-based model with Mahalanobis distance (ETMD)) [31] and generative adversarial one-shot diagnosis (GWOSD) [32] are used for comparison. For the feature extraction methods, the classifier SVM is identical. In detail, WPE and WPEE extract the energy and energy entropy of the signal components from each layer as features, respectively, and thus, 2^1^ + 2^2^ + 2^3^ + 2^4^ = 30 features are obtained. For MiniRocket, its univariate version with ten thousand kernels is adopted. For deep learning-based methods, end-to-end training is performed, and the default parameters from their paper are adopted.

It is worth noting that due to the temporal local nature, TLCE requires ten or two dozen times more data than MFCE to obtain competitive performance. Therefore, TLCE is not involved in the comparison.

**Figure 3:**
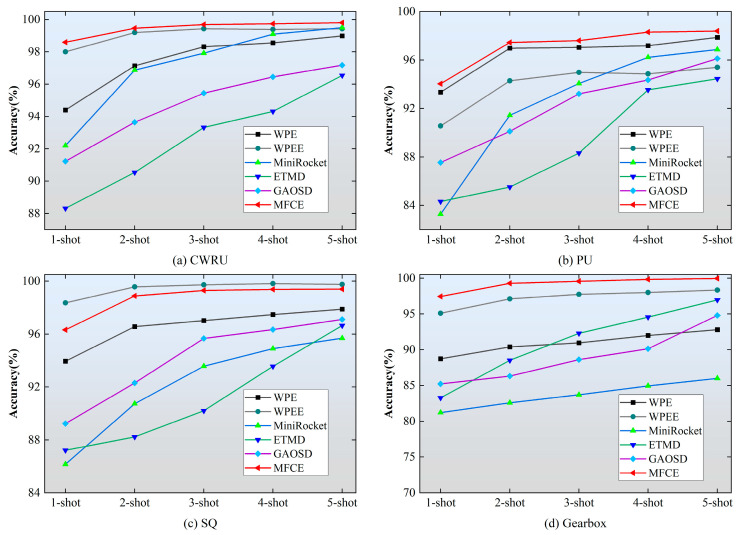
Comparison results of all methods.

Figure 3 shows the comparison results of all methods across different datasets in one-to five-shot settings. It can be seen that the average accuracies of MFCE are more than 98% in the five-shot setting; therefore, the proposed method is very suitable for real-world applications due to its excellent performance under limited-data situations. Although WPEE outperforms MFCE by a narrow margin on the SQ dataset, MFCE is significantly better than WPEE on the other three datasets by a much larger margin. It can also be noticed that MFCE is considerably better than ETMD and GAOSD on all datasets. Based on the tendency of ETM and GAOSD, they need more training samples to obtain the same accuracies as MFCE. In other words, MFCE is significantly better than deep learning-based methods under small sample sizes.

### 3.3. Noise Immunity Robustness Analysis

In real industrial scenarios, most of the collected signals contain lots of noise; therefore, so the immunity and robustness of MFCE in noisy environments are investigated. The experiment is implemented by adding Gaussian white noise with different signal-to-noise ratios to the original signal. The signal-to-noise ratio is defined as follows:

(14) $$SNR=10lg(P_{signal}/P_{noise})$$

where $P_{signal}$ and $P_{noise}$ denote the power of the original signal and the added noise, respectively.

**Figure 4:**
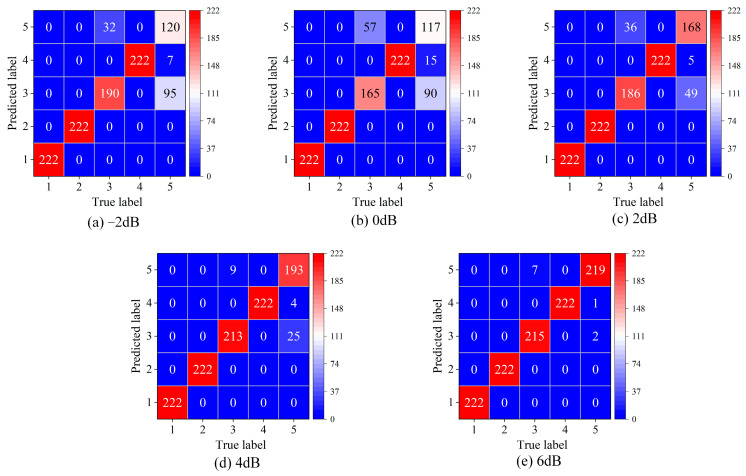
Confusion matrices of MFCE under different SNRs on the Gearbox dataset.

Figure 4 shows the confusion matrices of MFCE under different SNRs on the Gearbox dataset. It can be found that the number of misclassified samples decreases significantly with the increase in SNR. Table 3 shows the experimental results of all methods on all datasets, where the samples contain −2 to 6 dB noise in the five-shot setting. MFCE achieves an average accuracy of 89.33% at SNR = −2 dB and 92.34% at SNR = 0 dB, which are lower than the respective accuracies of 92.94% (WPE) and 94.79% (WPEE). However, MFCE attains an average accuracy of 95.24%, 97.50%, and 98.57% at SNR = 2, 4, and 6 dB, which surpasses other methods. This indicates that MFCE has advantages in environments with mild noise (SNR > 2 dB).

### 3.4. Parameter Analysis

In this section, the effects of the critical parameters, including the number of decomposition layers *K* and the wavelet basis function, are analyzed. Since the number of features extracted by MFCE is $4(4^{K}-1)/3$, the number of decomposition layers directly affects the performance of MFCE. Table 4 demonstrates the average accuracies of MFCE with different *K* values under the five-shot setting. The results show that the average accuracies of MFCE are 76.36%, 93.77%, 98.10%, 99.66%, and 99.76% when *K* ranges from 1 to 5. This indicates that by increasing the number of decomposition layers, MFCE can effectively improve its diagnostic performance. According to Equation (9), the computational complexity of the BBCM under the *k*th layer is *O*(4*^k^*). Therefore, the computational complexity increases exponentially as *K* increases. Considering the impact of the *K* value on the diagnosis accuracy and computation time, 4 is viewed as a reasonable *K* value.

**Figure 5:**
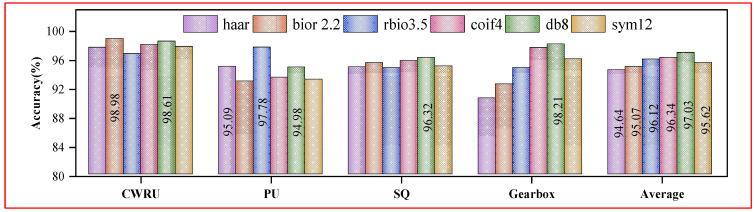
Accuracies of MFCE with different wavelet basis functions in the one-shot setting.

The wavelet basis function can capture specific frequency components of the signal, and thus, it has an essential influence on the performance of MFCE. Here, six typical wavelet basis functions are discussed, including the Haar wavelet, Biorthogonal (bior) 2.2 wavelet, Coiflets (coif) 4 wavelet, Reverse bior (rbio) 3.5 wavelet, Daubechies (db) 8 wavelet, and Symlets (sym) 12 wavelet. From Figure 5, the best performance on the SQ and Gearbox datasets is db 8. The best performances on the CWRU and PU datasets are bior 2.2 and rbio 3.5, with db8 ranked 2nd and 3rd, respectively. In general, db8 has a higher average accuracy than the others, so it can be regarded as a reasonable wavelet basis function.

### 3.5. Ablation Analysis

In this section, ablation analysis is carried out to demonstrate the rationality and superiority of MFCE further. To extract more correlation features, data expansion based on WPD is the most crucial part of MFCE. In addition to WPD, there are a large number of signal analysis methods capable of decomposing signals into signal components, including the empirical wavelet transform (EWT) [33], variational mode decomposition (VMD) [34], and Fourier decomposition method (FDM) [35], Ranumagin Fourier decomposition method (RFDM) [36]. These methods are integrated into MFCE to replace WPD for a fair comparison.

It is assumed that these methods decompose the original signal into *K* components, and the correntropy matrix between these *K* components and the original signal is computed to generate the (*K* + 1) × (*K* + 1) features. After selecting the best *K*, the corresponding accuracy is recorded. Experiments under the one-shot setting are carried out, and the results are shown in Table 5. It can be seen that MFCE achieves an accuracy of 94.98%, and the FMD achieved an accuracy of 99.08% on the PU dataset. It indicates that the data expansion by the signal analysis method is reasonable and effective. In addition, the average accuracy of MFCE (97.03%) is higher than that of EWT (52.90%), VMD (86.32%), FMD (94.82%), and RFMD (80.65%). It means that the introduced WPD is superior to other signal analysis methods.

## 4. Conclusions

In this paper, an MFCE combined with an SVM is proposed for the fault diagnosis of mechanical equipment. The results on the four datasets show that the MFCE is effective and robust.

The high-level diagnostic performance under small sample sizes demonstrates that the MFCE can effectively overcome the sample scarcity issue for model training. The excellent anti-interference performance demonstrates that the MFCE has good robustness in noisy scenarios.

In practical engineering, maintenance engineers and technicians in the field of mechanical systems can integrate the MFCE and SVM into health monitoring systems to monitor mechanical equipment in real-time and diagnose potential faults. In addition, necessary maintenance activities can be scheduled immediately upon the detection of faults in the equipment. In addition, mechanical equipment often experiences time-varying speed conditions, such as the start-stop process; therefore, the application of MFCE under time-varying speed conditions will be explored in the future.

## Figures and Tables

**Table 1:** Dataset description.

Information	CWRU	PU	SQ	Gearbox
Sampling rate (kHz)	12	64	25.6	5.12
Rotational speed (r/min)	1730	900	3000	1200
Number of fault types	10	8	7	5
Total sample size	117 × 10	42 × 8	56 × 7	227 × 5

**Table 2:** Accuracies and required training data of TLCE and MFCE.

Method	CWRU	PU	SQ	Gearbox	Average
TLCE	98.09%	93.27%	91.65%	97.48%	95.12%
	1024 × 13	6000 × 23	4600 × 16	3000 × 30	\
MFCE	98.60% (0.51% ↑)	94.98% (1.71% ↑)	96.32% (4.67% ↑)	98.21% (0.73% ↑)	97.03% (1.91% ↑)
	1024 (92.31% ↓)	6000 (95.65% ↓)	4600 (93.75% ↓)	3000 (96.67% ↓)	\(94.60% ↓)

The first and second rows represent the accuracies and required training data on the one-shot setting, respectively. ↑ represents the accuracy improvement compared with TLCE, while ↓ represents the percentage of data reduction compared with TLCE.

**Table 3:** Comparison results of all methods under different SNRs in the five-shot setting.

SNR (dB)	Method	CWRU	PU	SQ	Gearbox	Average
−2	WPE	94.86	94.70	96.59	85.61	**92.94**
	WPEE	91.89	91.86	97.45	87.84	92.26
	MiniRocket	82.02	85.45	85.45	80.15	83.27
	ETMD	80.21	79.21	84.56	84.30	82.07
	GAOSD	80.12	81.05	80.23	85.32	81.68
	MFCE	83.02	93.98	94.92	85.41	89.33
0	WPE	96.41	97.27	97.27	88.10	94.76
	WPEE	95.35	94.68	98.06	91.05	**94.79**
	MiniRocket	89.49	87.48	87.87	80.85	86.42
	ETMD	87.67	84.56	88.32	85.88	86.61
	GAOSD	88.21	83.23	89.32	87.56	87.08
	MFCE	89.33	95.32	96.77	87.93	92.34
2	WPE	96.69	97.47	97.32	88.99	95.12
	WPEE	95.57	95.20	98.18	91.67	95.16
	MiniRocket	95.22	89.86	90.59	81.77	89.36
	ETMD	90.34	88.82	92.22	93.40	91.20
	GAOSD	91.23	88.11	93.56	93.45	91.59
	MFCE	95.27	95.68	98.12	91.89	**95.24**
4	WPE	98.40	97.61	97.82	90.59	96.11
	WPEE	98.68	96.02	99.10	96.15	97.49
	MiniRocket	96.91	92.08	92.60	82.23	90.96
	ETMD	94.32	90.21	96.21	94.45	93.80
	GAOSD	95.11	90.34	95.33	95.21	94.00
	MFCE	97.78	96.80	98.82	96.58	**97.50**
6	WPE	98.59	98.34	97.85	91.44	96.56
	WPEE	99.32	96.26	99.61	97.22	98.10
	MiniRocket	98.60	93.66	93.73	83.16	92.29
	ETMD	95.67	93.21	97.21	96.45	95.64
	GAOSD	96.65	92.23	96.98	97.12	95.75
	MFCE	99.14	97.18	98.86	99.10	**98.57**

**Table 4:** Accuracies of MFCE under different numbers of decomposition layers.

The Number of Decomposition Layers	CWRU	PU	SQ	Gearbox	Average
1	61.39	82.47	80.79	80.78	76.36
2	96.72	96.96	90.57	90.82	93.77
3	99.54	97.05	97.92	97.87	98.10
4	99.84	98.93	99.93	99.92	99.66
5	99.95	99.23	99.94	99.91	99.76

**Table 5:** Accuracies of MFCE under different signal analysis methods in a one-shot setting.

Method	CWRU	PU	SQ	Gearbox	Average
EWT	45.70	54.82	47.77	63.32	52.90
VMD	86.91	84.31	91.64	82.43	86.32
FDM	95.45	99.08	94.22	90.53	94.82
RFDM	83.42	78.83	87.45	72.89	80.65
MFCE	98.60	94.98	96.32	98.21	97.03

## Data Availability

Data are available on request from the authors.
